# Accelerating measles and rubella elimination in southeast Asia

**DOI:** 10.1016/j.lansea.2025.100682

**Published:** 2025-10-10

**Authors:** The Lancet Regional Health – Southeast Asia

In August 2025, Nepal became the sixth country in southeast Asia to eliminate rubella as a public health problem. Home to a population of 29 million nestled between the towering Himalayas and difficult terrains, with a recently achieved federal status and a budding health system, this country's achievement is a testament to political will, multisectoral collaboration, and community engagement. We live in trying times for global health, as high-income countries are facing new outbreaks and rising mortality from previously eliminated diseases like measles. Global elimination efforts are largely being impeded by the rising antivaccination rhetoric, slashing of global funding, insufficient surveillance, and poor outbreak management. Amidst these challenges, Nepal's achievement offers a glimmer of hope.

The path to measles and rubella elimination in the southeast Asia region is being paved by smaller countries, inspiring larger nations to accelerate their efforts. With WHO extending the elimination target to 2026, time is of the essence. The recent meeting on verification of measles and rubella elimination by WHO concluded that bigger countries of the region—India, Bangladesh, Myanmar, and Thailand—are endemic for measles and rubella, and Nepal is still endemic for measles. Between 2000 and 2022, the WHO South-East Asia Region reduced its share of global measles deaths from 30% to 8%, and measles cases dropped by 86% from 12.4 million to 1.7 million. Following a decline during the COVID-19 pandemic, all endemic countries saw an increase in incidence of reported measles and rubella cases. From 2021 to 2023, India reported a surge from 4 to 45.3 measles cases and 1.2 to 2.1 rubella cases per million population. Thailand has also seen an increase in measles incidence (114 cases per million) and mortality in 2024.

Scientific evidence continues to affirm vaccines as a public health good with high return on investment, and the measles vaccine has had the highest impact in reducing infant mortality across regions and age groups. Most countries have improved vaccine coverage for measles and rubella post-pandemic, but subnational disparities persist, and uptake of the second dose continues to lag. On one hand, systemic factors contribute towards vaccine inequity by marginalising underserved populations such as refugees, migrants, homeless people, and other socially disadvantaged groups. On the other hand, there is increased vaccine hesitancy driven by cultural beliefs and misinformation. However, countries have made commendable efforts in closing the immunisation gap. India, for example, in addition to developing a strategic roadmap for measles and rubella elimination, has conducted multiple rounds of an immunisation campaign (known as Mission Indradhanush). It supplemented routine immunisation by targeting high-risk areas and children who missed scheduled doses of vaccine and has contributed to a significant improvement in immunisation coverage across the country. Bangladesh and Nepal conducted successful national supplementary immunisation, achieving more than 95% vaccination coverage.

Other strategies for elimination are also facing constraints. Surveillance in several countries is fragmented and ill established, relying on aggregate numerical reporting rather than detailed line-listing of individual cases. Recent outbreaks in countries such as Thailand have exposed the prevailing inequities, gaps in surveillance, lack of a systems approach, and insufficiencies in outbreak preparedness. Social indicators such as education and women's empowerment, known to result in high vaccine reach, are showing slow progress in the region. Cultural beliefs in disease causation and traditional treatments are derailing progress and perpetuating delays in health seeking. The existing system caters to paediatric surveillance of measles, while adult cases exacerbated by immunisation gaps are largely ignored. Countries need to confront their challenges and be open to divulge and introspect on indicators rather than resorting to biased reports highlighting only achievements. Recent policy change in India regarding integration of the WHO polio surveillance network—which was also catering to measles and rubella—into the country's national programme for surveillance signals a positive self-reliance by the government. But the proposed budgetary cuts, along with a hurried and unstructured transition, could also adversely impact surveillance activities.

We need to look back to move forward. From smallpox to polio, global cooperation and political commitment were key factors when it came to elimination. Polio elimination in the region offers replicable lessons. Although vaccine acceptance was aided in part by the oral route of administration and minimal side effects, the reasons for success extended far beyond pharmacological convenience. The supplementary immunisation campaigns, where vaccines reached door to door, were a key driver for success. The strategy yielded transformative outcomes: vaccines reached all parts of population equitably; intense advocacy efforts mobilised public awareness; diverse communities were actively engaged; and high-risk regions were systematically mapped and prioritised. Above all, it was the collaborative effort of multiple stakeholders and the commitment of grassroots health workers that turned the vision into reality.

In an era of accessible and affordable prevention, the persistence of measles-related deaths and congenital rubella syndromes reflects a troubling gap in political will to achieving health equity, undermining progress towards universal health coverage. To transform measles and rubella elimination into a systemic community-led movement, countries need to upscale the existing strategies backed up by local fiscal investments, intensively fight misinformation and myths by engaging and empowering communities, build sustainable systems, forge partnership with all agencies, and rely on data-driven decision making. A world where no child suffers from a preventable illness is within reach, but it demands urgent, coordinated, and scaled-up efforts.

